# Dynamical properties induced by state-dependent delays in photonic systems

**DOI:** 10.1038/ncomms8425

**Published:** 2015-06-17

**Authors:** Jade Martínez-Llinàs, Xavier Porte, Miguel C. Soriano, Pere Colet, Ingo Fischer

**Affiliations:** 1 Instituto de Física Interdisciplinar y Sistemas Complejos, IFISC (UIB-CSIC), Campus Universitat Illes Balears, E-07122 Palma de Mallorca, Spain

## Abstract

In many dynamical systems and complex networks time delays appear naturally in feedback loops or coupling connections of individual elements. Moreover, in a whole class of systems, these delay times can depend on the state of the system. Nevertheless, so far the understanding of the impact of such state-dependent delays remains poor with a particular lack of systematic experimental studies. Here we fill this gap by introducing a conceptually simple photonic system that exhibits dynamics of self-organised switching between two loops with two different delay times, depending on the state of the system. On the basis of experiments and modelling on semiconductor lasers with frequency-selective feedback mirrors, we characterize the switching between the states defined by the individual delays. Our approach opens new perspectives for the study of this class of dynamical systems and enables applications in which the self-organized switching can be exploited.

## Abstract (web summary)

Time delays in feedback loops and connections in dynamical systems and complex networks can depend on the state of the system, but these state-dependent delays are poorly understood. Here, the authors use a photonic system to characterize the switching between two loops with different delay times.

## Graphical abstract

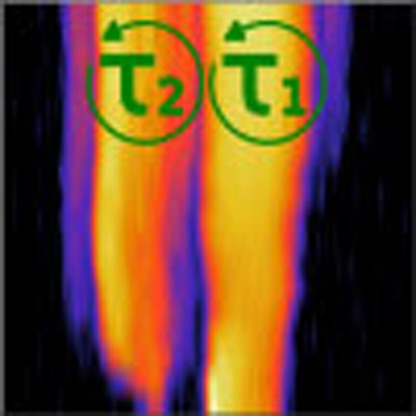

When the propagation time of a signal cannot be neglected, the resulting propagation delays can modify the properties of dynamical systems markedly, often giving rise to complex behaviour^1^. In many systems including internet traffic^2^, space communication^3^, control theory^4^, economics^5^, turning processes^6^, deep drilling^7^, predator–prey systems^8^ and blood flow 9, these delay times can depend on the state of the system. Moreover, also neural systems might show state-dependent distributions of conduction delay times^10^. Consequently, models of dynamical systems with state-dependent delay found considerable interest in mathematics and control theory, despite the fact that they are demanding to tackle^3^,^11^,^12^. State-dependent delays impose challenging problems in the mathematical analysis of the equations, as well as the numerical determination of solutions. Despite of the relevance and interesting properties of state-dependent delay systems, up to today hardly any experimental implementations and studies of such systems exist. Recently, an implementation of Boolean phase oscillators programmed within a FPGA was reported^13^. The state-dependent delay there played mainly the role to vary the effective coupling strengths.

Here we present a real-world photonic implementation of a dynamical system experiencing two different delay times depending on the state of the system. Although the system belongs to a conceptually simple class of systems with only two discrete delay times, not much is known about their dynamical properties, in particular in the chaotic domain. We employ a semiconductor laser system with two delay loops which are active depending on the laser's dynamical state. For not too strong pumping of the laser, persistent state-dependent switching dynamics between the different delay states is identified, corresponding to time intervals in which the feedback is mainly coming from one of the two delay loops. This switching dynamics is characterized in experiments and modelling on semiconductor lasers with frequency-selective feedback mirrors. On the basis of this work, new perspectives for the study of this class of dynamical systems arise, including their fundamental understanding, as well as their potential applications.

## Results

### State-dependent delay scheme

**Figure 1:**
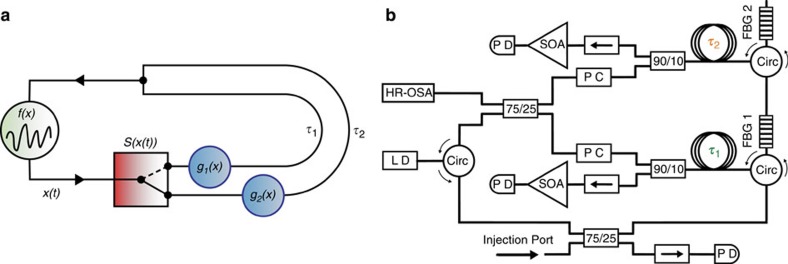
State-dependent delayed feedback scheme and its experimental implementation. (**a**) State-dependent delayed feedback scheme. An oscillator described by function *f*(*x*) and dynamical variable *x*(*t*) is directed to one or another branch via a switch depending on the state *x*(*t*). The two feedback branches are described via functions *g*_1_(*x*) and *g*_2_(*x*) and delay times *τ*_1_ and *τ*_2_, respectively. (**b**) Corresponding experimental configuration. LD: laser diode, Circ: optical circulator, PC: polarisation controller, 75/25 and 90/10: two by two optical couplers, FBG: fibre Bragg grating, →: optical isolator, SOA: semiconductor optical amplifier, PD: photodiode, and HR-OSA: high-resolution optical spectrum analyser. The two external cavities delays (*τ*_1_ and *τ*_2_) are schematically represented by the fibre spools after the respective FBG reflection. The fibre port labelled as *Injection Port* has been used to inject trains of short pulses to precisely measure the deskew and round trip times of the different external cavities.

A simplified scheme of the considered configuration is depicted in Fig. 1a. It comprises an oscillator with its dynamics described by a variable *x*(*t*). Using a state-dependent switch, depending on *x*(*t*) the dynamics is routed to either one or another feedback branch with corresponding functions *g*_1_(*x*) and *g*_2_(*x*) and delay times *τ*_1_ and *τ*_2_, respectively. The scheme belongs to a class of state-dependent delay systems described in general by a vector variable **x** following:


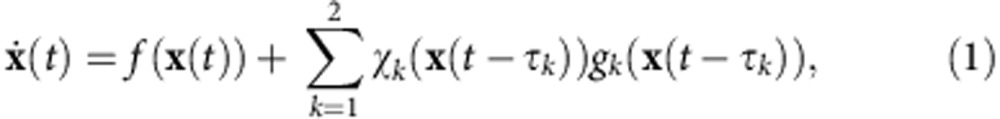


with χ_1_(***x***(*t*)), χ_2_(***x***(*t*))∈{0,1} and *χ*_1_(***x***(*t*))·χ_2_(***x***(*t*))=0. Therefore, the switch *S*(*x*(*t*)) is represented here by the functions χ_1_(***x***(*t*)), χ_2_(***x***(*t*)). It is worth noting that, due to the position of the switch, at any moment the oscillator obtains feedback either from branch 1, from branch 2, from both branches or from none of them. Configurations with the switch after the delay loops would result in different behaviour. Moreover, this represents only one of the simplest configurations of many state-dependent delay feedback or coupling motifs that can be imagined and realized in the future.

### State-dependent delay experiment

Our experimental implementation is based on the well-studied semiconductor laser systems with delayed optical feedback (see for example refs 14, 15 and references therein). Here, however, we replace the common mirror or fibre loop with fixed delay by two feedback loops with mutually exclusive spectral reflection properties and different lengths, resulting in different delay times. Theoretically, those systems were recently studied in terms of their relative equilibria structure and stability^16^,^17^. In a recent experiment a second frequency-selective feedback loop was introduced to control the frequency oscillations of the laser light^18^. Here we consider the limit that both delays are long compared with the characteristic dynamical time scale of the laser. Specifically, semiconductor lasers exhibit a characteristic amplitude–phase coupling^19^, therefore amplitude dynamics is usually associated with dynamics of the optical frequency as well. This allows us to act on the spectral characteristic of the state rather than its amplitude, since amplitude-related nonlinearities typically only give rise to very small relative changes of the delay times. It is more convenient and very flexible to choose different delay loops depending on the corresponding optical spectrum of the laser emission by employing spectrally selective (filtering) mirrors.

For our experiments, we have employed a discrete-mode quantum-well semiconductor laser emitting at 1543, nm with a threshold current of *I*_*th*_=12.00 mA^20^. With and without external optical feedback, the discrete-mode laser exhibits single-mode behaviour with a side-mode suppression ratio larger than 35 dB and a longitudinal mode separation of 150 GHz. We have measured a linewidth enhancement factor *α*∼2 using the Henning–Collins approach^21^. The current and temperature of the laser are stabilised to an accuracy of 0.01 mA and 0.01 K, respectively.

Figure 1b depicts the experimental setup, employing standard telecommunications components. The laser is subject to filtered optical feedback (FFB) from two distinct cavities of different length and with disjunct spectral reflection ranges. Each feedback cavity contains an independent fibre-loop mirror closed by a fibre Bragg grating (FBG) that acts as the frequency-selective reflector. The reflection bandwidths of the FBG have been determined to be 4.63±0.02 GHz for FBG1 and 5.69±0.02 GHz for FBG2. The respective centre frequencies of FBG1 and FBG2 have been detuned −4±0.01 GHz and −11±0.01 GHz with respect to the solitary laser frequency (see Methods section). Therefore, there is no spectral overlap between the reflection bandwidths of the two FBGs. Polarisation controllers are used to independently align the polarisation for each filtered-feedback cavity. The fibre transmission within the delay loops is considered as linear propagation. It is important to note that the laser represents the nonlinear element, and in combination with the FBGs acts as the state-dependent switch, as will be discussed in the context of the experimental and modelling results.

The design of the experimental setup allows for direct detection of the light emitted from the laser diode, and moreover of the light reflected from each individual FBG. About 25% of the light emitted by the laser diode is coupled out for detection, while for the light reflected from each FBG this fraction amounts to only 10%. Therefore, for measuring the FBG reflected light, two semiconductor optical amplifiers are employed to amplify the signals before detection. Via the last coupler in the feedback line, 75% of the light from FBG2 and 25% of the light from FBG1 are merged and fed back to the laser diode, resulting in an effective ratio of feedback strengths of 4.38 dB. The second output port of the coupler is used for detection of the optical spectrum. The measurements of the dynamics were performed using a 13 GHz bandwidth detector for the total intensity dynamics and two 20 GHz detectors for the filtered dynamics and using a 16 GHz analogue bandwidth oscilloscope with a sampling rate of 40 GS s^−1^.

We have precisely measured the external cavity round trip times and the relative deskews between detection channels by injecting 1 ns light pulses into the injection port (see Fig. 1b). The pulses have been wavelength—tuned to the centre frequency of the FBG to be measured. With this procedure, we have measured the delay times of the two cavities as *τ*_1_=106.075±0.025 ns and *τ*_2_=121.625±0.025 ns.

### Experimental results

**Figure 2:**
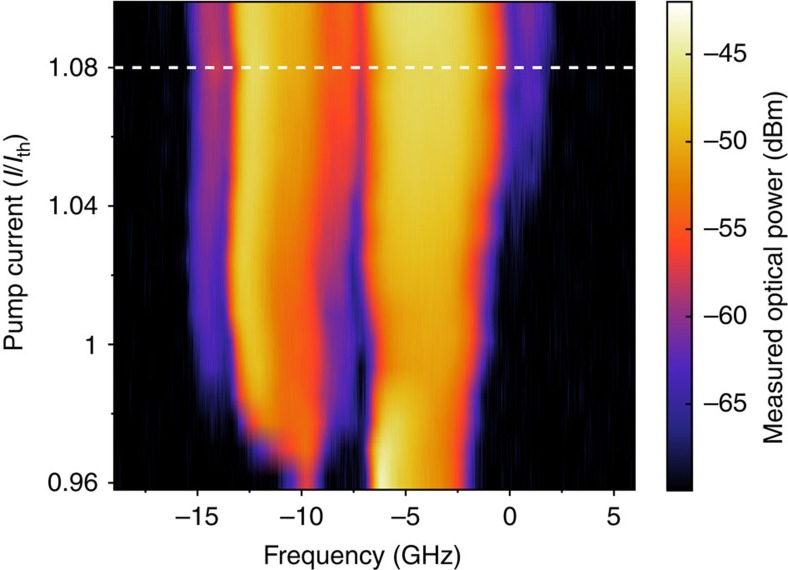
Dependence of the optical spectrum on the pump current of the laser. The frequency axis is centred to the value of the solitary laser frequency (194,350 GHz). FBG1 and FBG2 are detuned from the solitary laser frequency by −4±0.01 GHz and −11±0.01 GHz, respectively. The dashed horizontal line indicates the highest current for which the two spectral bands corresponding to the filters are separated by a gap larger than 10 dB. The relative amplitudes of the two spectral components are affected by the coupling ratio of the coupler at which the spectra are measured.

Figure 2 exhibits optical spectra of the laser diode emission depending on the injection current. Since the feedback from the external reflectors reduces the threshold current of the laser, the optical spectra are depicted for pump currents ranging from 4% below the solitary laser threshold to 10% above. For currents below 1.08*I*_*th*_, the two filters can be considered isolated, with a spectral gap >10 dB between them (indicated via a horizontal dashed line in Fig. 2). For pump currents above 1.08*I*_*th*_, this gap gradually disappears and the dynamics resulting from these spectral signatures cannot be considered spectrally separated anymore. We note that the spectra close to threshold exhibit comparable spectral amplitudes and bandwidths in both filters and, therefore, one can expect similar contributions to the dynamics from both filters. Therefore, due to the chosen geometry, and for sufficiently low pump current, our system belongs to the class of systems described by the state-dependent delay equation 1.

**Figure 3:**
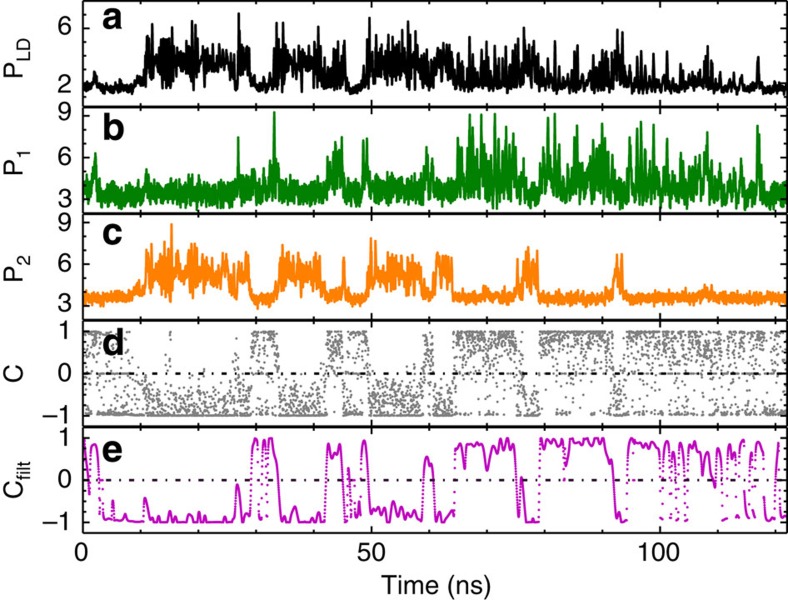
Experimental time series for the pump current of *I*=1.01*I*_*th*_: (**a**) intensity dynamics emitted by the laser; (**b**,**c**) filter-resolved intensity dynamics for FBG1 and FBG2, respectively; (**d**) contrast function; and (**e**) contrast function of the 1 GHz low-pass filtered intensity dynamics. Intensities have been plotted in arbitrary units.

Figure 3 depicts the time series of the laser emission and the intensity in the two respective feedback loops for a pump current of *I*=1.01*I*_*th*_. The total intensity dynamics is depicted in Fig. 3a. Figure 3b,c show the filter-resolved dynamics for FBG1 and FBG2, respectively. These latter time series demonstrate the state-dependent nature of the dynamics, with alternating periods of emission in the frequency bands corresponding to FBG1, FBG2 or in none of both filters resulting in no feedback. The complementary nature of the total dynamics based on mostly exclusive contributions either from FBG1 or FGB2 is already visible by eye. To quantify this further, the signature of this state-dependent delay dynamics is visualised in Fig. 3d,e using a contrast function defined as 
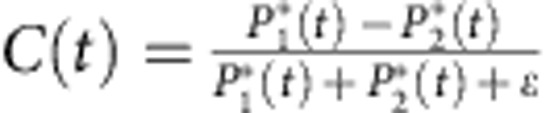
 with 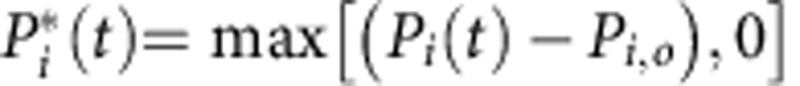, *i*=1, 2 and *ɛ* being infinitesimal. This definition of the contrast function is based on the careful characterisation of the origin of noise in the detected time series. We can clearly attribute the origin of noise to detection noise, which is symmetric around the actual physical value. Therefore, we subtract an offset *P*_*i*,*o*_ which has been determined based on this characterisation. The infinitesimal parameter *ɛ* is introduced to avoid indeterminations of the form zero divided by zero, since both powers *P*_i_* can become zero simultaneously. Figure 3d,e depict the contrast functions obtained from the fully resolved and the 1 GHz low-pass filtered intensity time traces, respectively. From both time traces of the contrast function, the switches between the states can be clearly recognised. In the contrast function in Fig. 3d, one can see also the fluctuations due to the influence of detection noise. Those fluctuations do not represent actual switches between states and are mostly removed by the low-pass filtering, giving rise to the much smoother contrast function in Fig. 3e. To further characterize the switching properties between the two filter states and the zero-feedback state, we have calculated the cross-correlation coefficient between *P*_1_ and *P*_2_. We obtain *C*_*c*_=−0.4 for the unfiltered time traces and *C*_*c*,*f*_=−0.65 for the filtered ones, supporting that the intensity dynamics in the two filters is anticorrelated.

**Figure 4:**
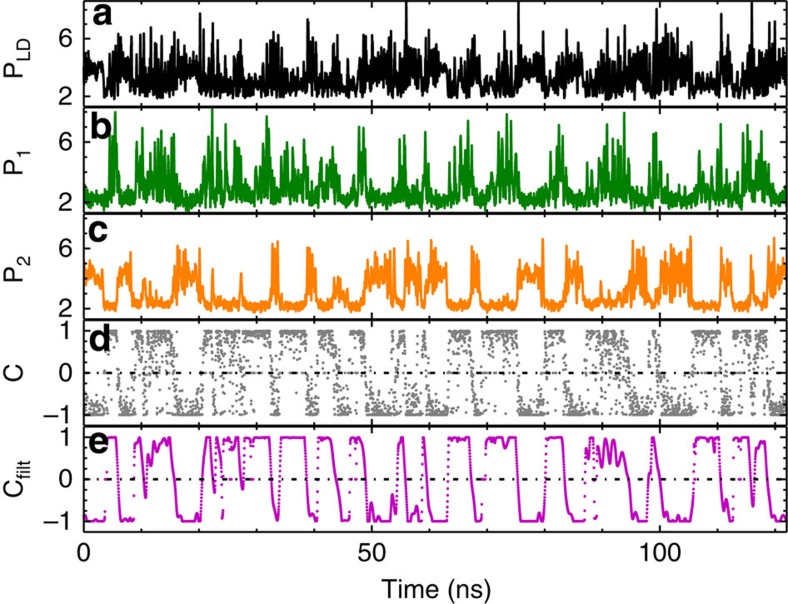
Experimental time series for the pump current of *I*=1.07*I*_*th*_: (**a**) intensity dynamics emitted by the laser; (**b**,**c**) filter-resolved intensity dynamics for FBG1 and FBG2, respectively; (**d**) contrast function; and (**e**) contrast function of the 1 GHz low-pass filtered intensity dynamics. Intensities have been plotted in arbitrary units.

Increasing the injection current results in dynamics exhibiting faster transitions between the different state-dependent delays (filter states) and shorter characteristic residence times in each state. This is illustrated in Fig. 4, showing the state-dependent delay dynamics for an injection current of *I*=1.07*I*_*th*_. The contrast function again indicates clear switches between the different filter states, providing evidence for state-dependent delay dynamics. Increasing the injection current farther, results in an increasing bandwidth of the laser dynamics, related to the increase of the relaxation oscillations frequency. This ultimately results in a spectral overlap as illustrated in Fig. 2 and therefore in the loss of a clear distinction between two separate states. Nevertheless, for pump currents below *I*=1.08*I*_*th*_ the laser indeed acts as nonlinear element and in combination with the FBGs as state-dependent switch, determining which delayed feedback loop will be chosen. Before discussing the statistics of the switchings between different filter states in more detail, we introduce a model for the system using a rate equation approach, and compare its properties with the experiments.

### Numerical modelling

We consider a simple model for a semiconductor laser subject to the feedback from *m* frequency-selective external cavities. The dynamics is analysed through the extended Lang–Kobayashi^22^ model that consists of 2+*m* equations in its simplest form. The first two, for the slowly varying complex amplitude of the electric field *E*(*t*) (in the reference frame of the solitary laser frequency) and the carrier number *N*(*t*), are given by


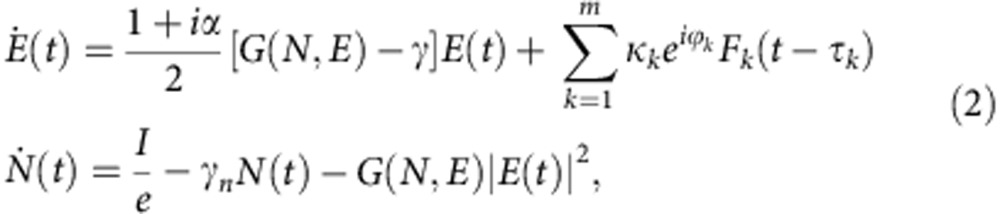


where *F*_*k*_(*t*) is the slowly varying complex amplitude of the filtered electric field that is fed back to the laser after spectral filtering from filter *k*, *α* is the linewidth enhancement factor, *γ*=0.2 ps^−1^ is the photon decay rate, *γ*_*n*_=1 ns^−1^ is the decay rate for the carriers, *I* is the injection current, *e* is the electron charge and *τ*_*k*_, *κ*_*k*_ and *ϕ*_*k*_ are the feedback delay time, the feedback strength and the accumulated phase of filter *k*, respectively. In the long-cavity limit, as considered here, the phases do not play a significant role^14^, so we choose *ϕ*_*k*_=0. The Lang–Kobayashi approach is valid for a single longitudinal mode of the internal cavity. We consider a nonlinear gain 
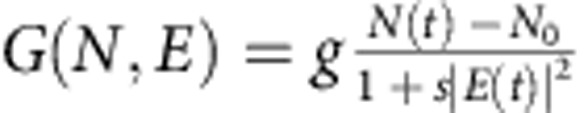
, where *g*=1.5 × 10^−8^ ps^−1^ is the differential gain coefficient, *N*_0_=1.8 × 10^8^ the carrier number at transparency and *s*=10^−7^ the gain saturation coefficient. The threshold current of the solitary laser, *I*_*th*_=*eγ*_*n*_(*N*_0_+*γ*/*g*), is 30.98 mA. For convenience we define the adimensional carrier number as *n*=(*N*−*N*_0_)*g*/*γ*−1.

For ease of analytic and numerical tractability, we consider Lorentzian linear filters with centre frequency Ω_*k*_ and half width at half-maximum Λ_*k*_^23^,^24^. Then, the extended Lang–Kobayashi equation (2) are completed with *m* equations, one for each filter,


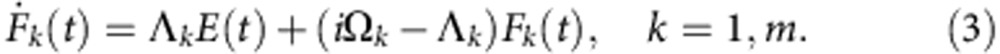


The aim of this approach is to provide a simple model that allows for qualitative comparison with the experiments and for a detailed study of the underlying mechanisms. Precise quantitative agreement between theory and experiment cannot be expected, since the Lorentzian filter profile does not reflect the detailed filter characteristics of the fibre Bragg gratings. Nevertheless, we can choose similar parameters, which give rise to similar physical conditions and dynamical behaviour.

The relative equilibria of the system equations (2)–(3), 6 are filtered cavity modes, FCMs, which are rotating wave solutions with constant frequency, carrier number and field amplitude. They can be calculated depending on laser and filter parameters, as detailed in the Methods section.

Mimicking the experimental conditions qualitatively, we have performed numerical simulations of (2)–(3) using different sets of parameter values. First, we consider a laser with a linewidth enhancement factor of *α*=3 and two cavities with *κ*_1_=*κ*_2_=40 ns^−1^, Λ_1_/(2*π*)=Λ_2_/(2*π*)=0.5 GHz, *τ*_1_=106.075 ns, *τ*_2_=121.625 ns, Ω_1_/(2*π*)=−5 GHz and Ω_2_/(2*π*)=−15 GHz, for several values of the injection current.

**Figure 5:**
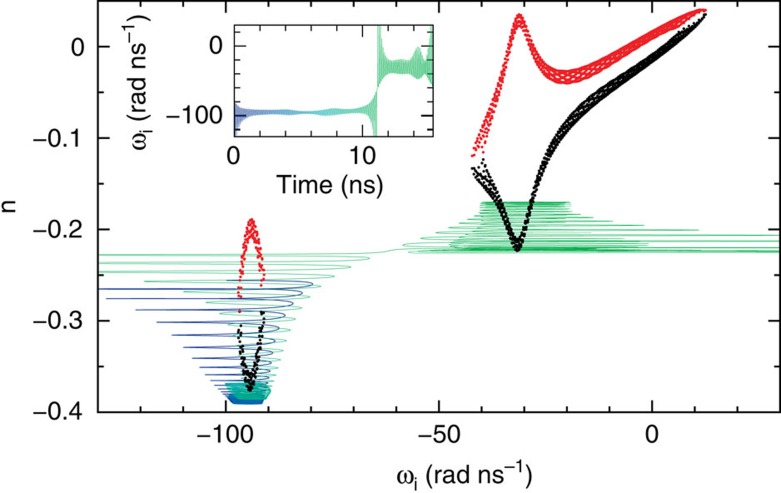
Numerically obtained trajectories. 15.5 ns trajectory of (2)–(3) with two filters around modes (black points) and antimodes (red points). The parameters are *α*=3, *I*=0.99*I*_*th*_, Ω_1_/(2*π*)=−5 GHz, Ω_2_/(2*π*)=−15 GHz, *κ*_1_=*κ*_2_=40 ns^−1^. The inset shows the instantaneous optical angular frequency as function of the time.

**Figure 6:**
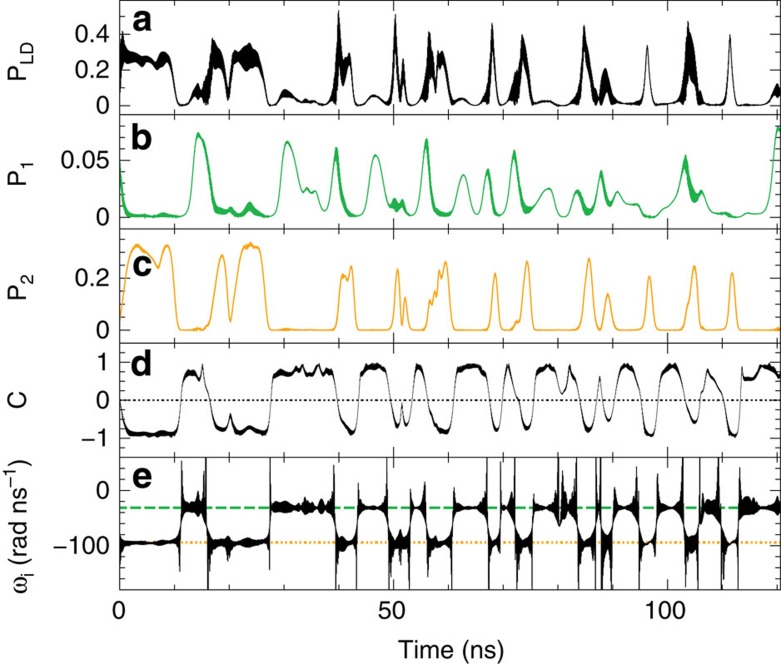
Numerical time traces. Numerical time traces for the same parameters as in Fig. 5: (**a**) intensity of the electric field, (**b**) *P*_1_(*t*)=|*F*_1_(*t*)|^2^, (**c**) *P*_2_(*t*)=|*F*_2_(*t*)|^2^, (**d**) filter contrast and (**e**) instantaneous optical angular frequency of the electric field. In the last panel, the dashed lines indicate Ω_1_ (green) and Ω_2_ (orange).

For injection currents sufficiently below threshold, *I*/*I*_*th*_<0.99, and neglecting noise in the simulations, the system gets trapped at the most stable FCM, with lowest *n* and frequency close to the centre frequency of the more detuned filter. For injection currents in the interval *I*/*I*_*th*_=0.99−1.1, the system exhibits switching dynamics between the two islands of FCMs, corresponding to the two solitary filters with different delay times. Remarkably enough, for large delays the switching regime can start even below the solitary laser threshold. As an example, Fig. 5 shows a 15.5 ns trajectory of the system for *I*/*I*_*th*_=0.99 together with the FCMs in the (*ω*_*i*_, *n*) plane. The corresponding instantaneous optical angular frequency *ω*_*i*_, relative to the solitary laser frequency, is plotted in the inset as function of time. In Fig. 5, one can recognise how the system jumps from a state centred in the more detuned filter (blue region) towards the less detuned one (green region). Despite of the tails of the filter functions, the FCMs of the system with two filters lie very close to the FCMs of a corresponding system with a single filtered feedback. One can therefore conclude that the dynamics indeed exhibits state-dependent delay properties. The instantaneous optical angular frequency of a larger trajectory with several jumps is shown as a function of time in Fig. 6e, together with the intensity of the electric field (a), the intensity of the filtered fields *P*_1_(*t*)=|*F*_1_(*t*)|^2^ (b) and *P*_2_(*t*)=|*F*_2_(*t*)|^2^ (c) and the filter contrast (d). In Fig. 6e, the centre frequencies of the two filters are plotted as dashed lines to illustrate the time intervals in which the trajectories oscillate around the relative equilibria of each filter. The time intervals during which the trajectories are concentrated in one of the filters are shorter than the delay times.

For injection currents *I*/*I*_*th*_>1.1, the dynamical evolution of the system converges to a global chaotic attractor, in which distinct jumps between the two different filters can no longer be identified. We have checked that for the configuration considered here the range of currents in which state-dependent dynamics is found in numerical simulations is robust against small changes in the parameter values.

**Figure 7:**
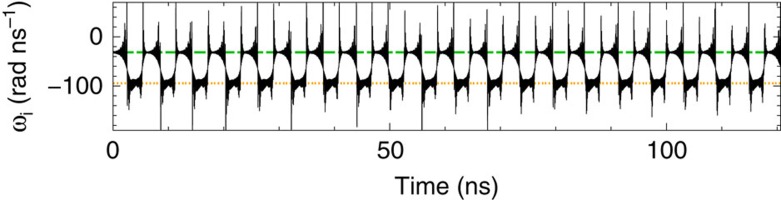
Instantaneous optical angular frequency of the electric field for *τ*_1_=30 ns and *τ*_2_=36 ns. The remaining parameters are the same as in Fig. 5.

In addition to the dynamical states reported above, close to threshold it is also possible to observe state-dependent dynamics in which the switchings between the two filter states take place at more regular time intervals. This regularity is further enhanced if the delay times are shorter. For instance, for delay times of 30 and 36 ns and pump currents around *I*/*I*_*th*_=0.99, one gets periodic switchings where the periodicity is close to the difference of the delay times. An example of such dynamics is depicted in Fig. 7. Similar dynamics with periodicity of the order of the difference in delay times is not restricted to specific ratios *τ*_1_/*τ*_2_, but can be found over small windows of ratios [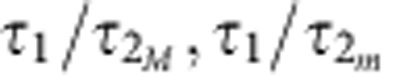] of size 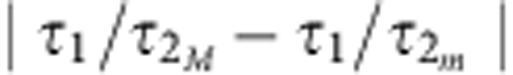 of the order of 10^−1^. This kind of dynamics resembles square wave behaviour, as also reported for switching among polarization modes in edge-emitting semiconductor lasers^25^,^26^, VCSEL^27^ and fibre lasers^28^, and between the lasing directions for semiconductor ring lasers^29^, among others. The peculiarity of the system considered here is twofold. First, the two states are not individual modes, but are dynamical states which can involve many external cavity modes. Second, the two states involved in the switching are associated with different delay times.

**Figure 8:**
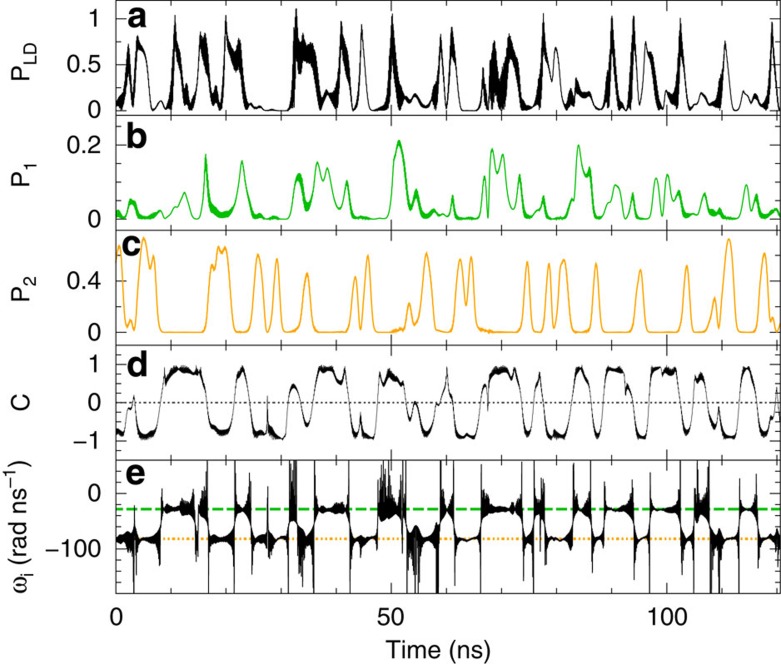
Numerical time traces. Numerical time traces for *α*=2, *I*=0.999*I*_*th*_, Ω_1_/(2*π*)=−4.5 GHz, Ω_2_/(2*π*) =−13 GHz, *κ*_1_=30 ns^−1^ and *κ*_2_=50 ns^−1^: (**a**) intensity of the electric field, (**b**) *P*_1_(*t*)=|*F*_1_(*t*)|^2^, (**c**) *P*_2_(*t*)=|*F*_2_(*t*)|^2^, (**d**) filter contrast and (**e**) instantaneous optical angular frequency of the electric field. In the last panel, the dashed lines indicate Ω_1_ (green) and Ω_2_ (orange).

We have performed systematic numerical studies to investigate the influence of key parameters on the dynamical behaviour. We find state-dependent delay dynamics for considerable parameter regimes covering intervals for the injection current of (*I*/*I_th_*=0.99−1.1), the studied range of *α*∈[2,3], filter detunings of ΔΩ/(2*π*)∈[−8,−12] GHz and some variation of the absolute filter positions by a few GHz. Choosing *α*=3, Ω_1_/(2*π*)=−5 GHz and Ω_2_/(2*π*)=−15 GHz, or *α*=2, Ω_1_/(2*π*)=−4.5 GHz and Ω_2_/(2*π*)=−13 GHz, we obtain state-dependent delay dynamics for *κ*_1_∈[30,50] ns^−1^ and *κ*_2_∈[40,50] ns^−1^ (|*κ*_1_−*κ*_2_|≤20 ns^−1^). Figure 8 depicts a situation nominally even closer to the experimental parameters with *α*=2, Ω_1_/(2*π*)=−4.5 GHz, Ω_2_/(2*π*)=−13 GHz, *κ*_1_=30 ns^−1^ and *κ*_2_=50 ns^−1^. The figure illustrates that one finds similar switching dynamics between the filter states as depicted in Fig. 6. The fact that we find state-dependent delay dynamics in a considerable parameter range and for different filter characteristics proves the robustness of the observed phenomena.

Finally, we note that if the filter configuration is changed such that the longer delay is assigned to the less-detuned filter, we find a larger pump current region close to threshold in which the system operates in a stable FCM. As a consequence, the region of state-dependent delay dynamics is smaller or can even become negligible in parameter space.

### Switching characteristics

**Figure 9:**
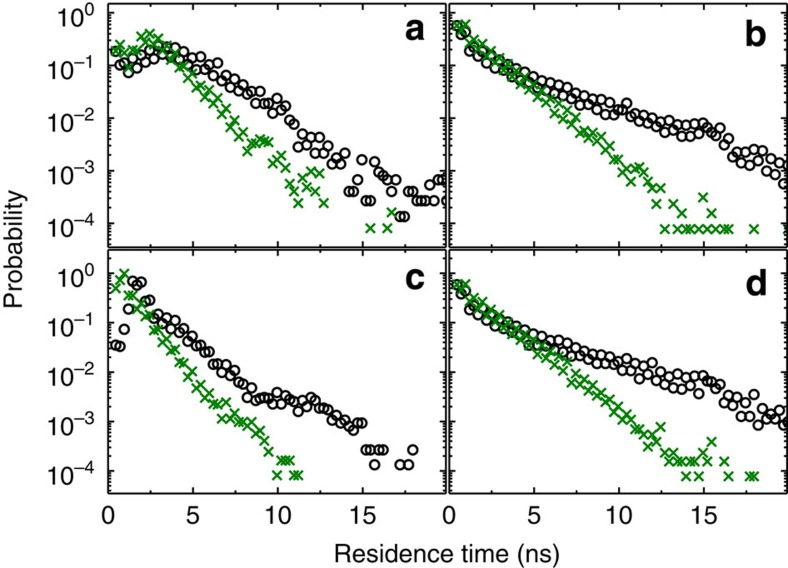
Residence-time distributions. Numerical, (**a**,**c**), and experimental, (**b**,**d**), residence-time distributions in the states corresponding to the two delays of *τ*_1_=106.075 ns and *τ*_2_=121.625 ns. Dots correspond to 1.0*I*_*th*_ and crosses to 1.1*I*_*th*_. (**a**, **b**) show the residence times in filter state 1 while (**c**,**d**) correspond to filter state 2.

In Fig. 9 we compare the residence-time distributions of experiment and modelling for two different pump currents, corresponding to 1.0*I*_*th*_ and 1.1*I*_*th*_. The residence times have been extracted from the contrast function for 195 μs long time traces. Before calculating the contrast function, the time series have been averaged over windows of 0.1 ns. The experimental time series have been low-pass filtered with a cutoff frequency of 1 GHz. This filtering removes the fastest noisy oscillations, unrelated to switches in delay states. In all panels one can recognise that the residence-time distributions in this irregular switching regime show a maximum of switchings on fast time scales of few nanoseconds or less. Moreover, the residence-time distributions approximately exhibit exponential tails that decay for times <20 ns. Two conclusions can be drawn from the comparison of experiment and modelling: first, for lower bias current the system exhibits higher typical residence times than for higher bias currents; and second, residence times longer than the difference between the two delay times (∼15 ns) are very rare. This can be understood when considering that switches between the two filter states, corresponding to the different delay times, can have two possible origins. They can either occur spontaneously because of the dynamics within one filter state, or they are induced by a previous filter jump being fed back into the laser. Such major perturbations will with a significant probability change the filter state and therefore the delay. Due to equation 1, these induced events will occur in intervals smaller than the larger delay time and even smaller than the difference in delay times. Spontaneous jumps turn out to be much less likely. Remarkably, irrespective of the different filter shapes in modelling and experiment, the residence-time distributions exhibit a similar decay. A deeper understanding of the residence-time distributions and their dependence on the various system parameters is left for future investigations.

## Discussion

The presented results demonstrate in experiment and modelling a photonic system with two delay times that displays a dynamical regime dominated by state-dependent delay dynamics. In this regime the system effectively switches between two well-defined delays determined by the state of the system itself. We have implemented the two delays as two fibre delay loops of different length, each with a frequency-selective Bragg filter. The two filters are centred at different frequencies and therefore the frequency of the optical field, associated to the state of the system, determines which of the two delays is active at a given time interval. In this system, the state-dependent delay dynamics results from the interplay between the laser bandwidth, the central frequencies of the filters and the bandwidth of the filters. In particular the overlap between the filters has to be sufficiently small in order to clearly distinguish the two states. From a theoretical point of view this means that the stationary rotating wave states (filtered cavity modes) of the overall system are basically located in two separated regions in phase space. The states in each region are similar to those that appear in a semiconductor laser with a single delayed feedback. We should also note that state-dependent delay dynamics has a different nature than the dynamics arising in systems with time dependent delays. In the latter ones, the delay time is externally modulated^30^ while in the former ones the effective delay is intrinsically determined by the state of the system which, in turn, depends on the delays active at each time interval. Therefore, in state-dependent delay systems, the changes in delay times emerge in a self-organized manner.

We have shown that in a semiconductor laser system with two filtered delayed feedback loops one encounters different dynamical regimes as the pump current is increased. State-dependent delay dynamics has been identified close to the laser emission threshold when the delay times are chosen such that the longer delay corresponds to the more detuned Bragg filter. Experimentally, we observe the existence of time intervals in which the feedback is mainly coming from one of the two filters and we have shown that the relative intensity difference is a suitable quantifier to identify state-dependent delay dynamics. From a theoretical point of view, these time intervals of feedback from a single filter correspond to trajectories in phase space which remain around the filtered cavity modes associated to a given delay loop. The observation of state-dependent delay dynamics requires that the jumps between the regions in phase space associated to the two delay loops do not occur too frequently. Increasing the pump leads to more frequent jumps so that the dynamics effectively mixes the two states of the system. In this case, experimentally one sees that for the vast majority of time, there is a significant amount of feedback coming simultaneously from both delay loops.

We emphasize that this is a well-controllable and reconfigurable experimental system built from standard telecom components in which the dynamics can be precisely measured. Thus, our approach represents a prototypical study of state-dependent dynamics and opens major opportunities to investigate different implementations and classes of state-dependent delay systems. One can, for instance, reverse the order of the filters, or increase the number of filters. Moreover, one can extend the studies from discrete delays to continuous dependences of the delay on the dynamical state by using chirped Fibre Bragg gratings, with either positive or negative chirp. For most of these situations, generic mathematical models exist that allow to fit the system in an appropriate general background. To obtain a more precise correspondence of experiment and modelling, one could extend the numerical investigations to travelling wave models, which would allow to take the FBG filter characteristics more accurately into account. Overall, our approach represents a flexible platform, that allows for a detailed experimental and theoretical exploration of state-dependent delay systems and their possible applications.

Direct measurement of the state-dependent delay dynamics in systems with controllable parameters is quite relevant for scientific fields well beyond optics. For instance, state-dependent delays play a critical role in the dynamical evolution of age-structured biological or physiological processes such as erythropoiesis^31^,^32^ and state-dependent population growth^33^. But also technological applications can be envisaged, exploiting the self-organized erratic switching between different delay states. In particular, key exchange and encryption systems could benefit from such switching. Recently, key exchange protocols have been introduced that utilize the synchronization of delay-coupled systems^34^,^35^. For some of them the irregular switching of conditions or filters has been suggested to enhance security^35^. Irregular jumps between states induced by state-dependent delay dynamics could play this role without the need of externally controlled switching. Also for control systems, the self-organized switching between different loops with different delays and potentially performing different transformations provides new possibilities. One could, for instance, implement different control loops depending how far one is from a target state, or allow for specific control loops for multiple possible states.

## Methods

### Fibre bragg gratings

The two FBG filters used in the experiments are grating structures of 1 mm length photoimprinted in standard single-mode fibre (SMF28). Their maximum reflectivities are >90% and the measured reflection bandwidths are 4.63±0.02 GHz and 5.69±0.02 GHz for FBG1 and FBG2, respectively. One advantage of employing FBGs is that they allow for tuning of their reflection frequencies without essential modification of any of their other characteristics, like reflectivity and reflection bandwidth. For this purpose, both FBGs have been fixed to independent translation stages in order to tune them to the desired reflection frequencies by means of stress. The frequency dependence on length changes is *δf*/*δL*=1.1±0.1 GHz/*μ*m, and the maximum tuning range is 250±60 GHz. In the presented experiments, the reflection bandwidths of the FBG, their spectral positions and relative amplitudes were precisely measured with the combination of a super-luminescent diode and a high-resolution optical spectrum analyser with 10 MHz resolution^36^.

### Relative equilibria of a semiconductor laser with Lorentzian filtered feedback

The dynamics of a semiconductor laser system with Lorentzian FFB from *m* filters is given by


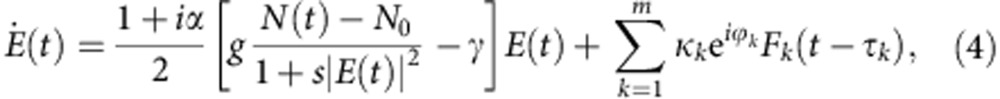



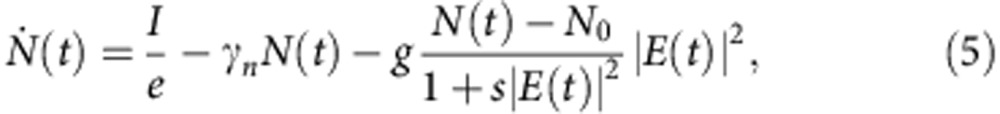



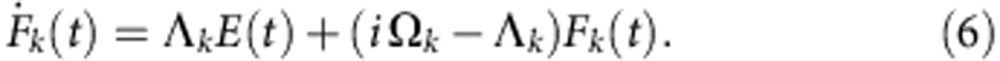


The relative equilibria of the system (4)–(6), 10, 11 are filtered cavity modes, FCMs, which are rotating wave solutions with constant frequency, carrier number and field amplitude,


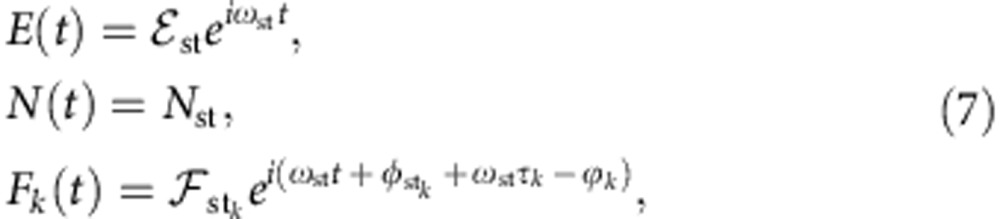


where 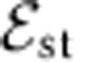, 
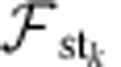
, *N*_st_, *ω*_st_ and 
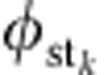
 are real positive constants. Inserting this ansatz into (4) and (5) yields


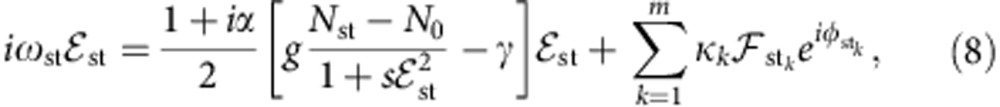



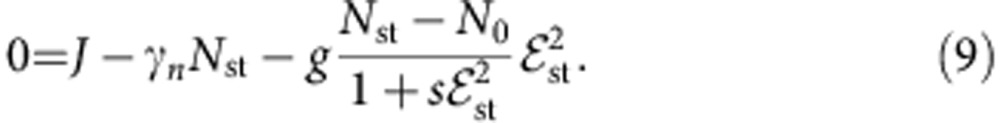


For Lorentzian filtering, inserting the ansatz (7) into (6), 11 gives rise to


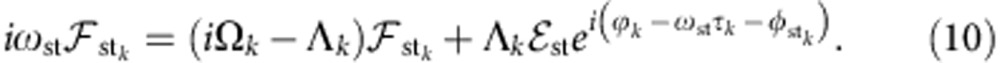


Real and imaginary part of equation (10),


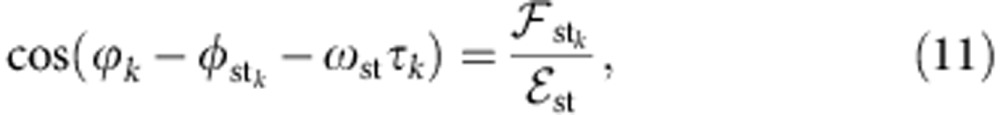



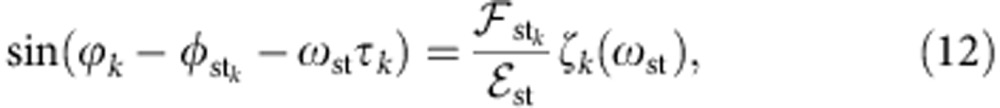


can be combined to get an equation for 
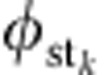
,


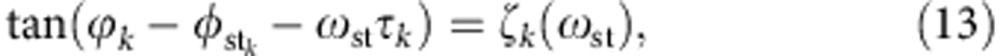


where,


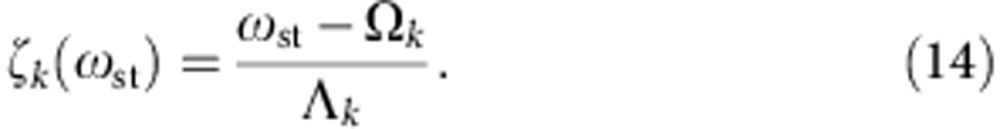


Since in equation (11) 
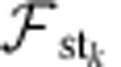
 and 
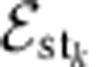
 are positive the argument 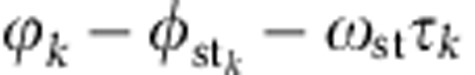 must be either in the first or in fourth quadrant. Then equation (13) is equivalent to


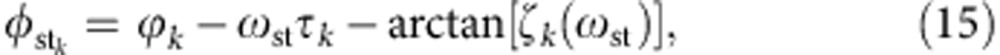


Substitution of (15) into (11) gives rise to


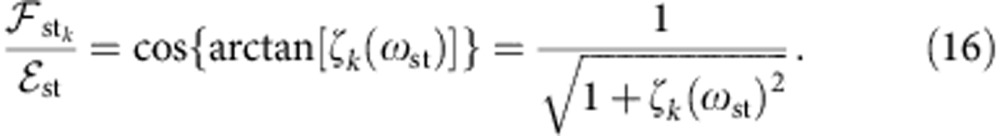


With the purpose to obtain an equation for *ω*_st_, we separate equation (8) into its real and imaginary parts, yielding


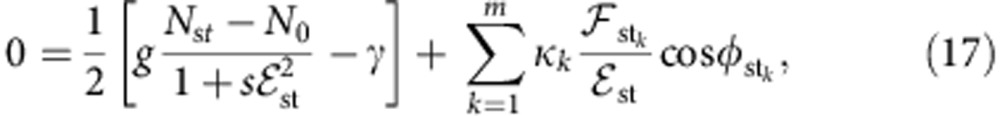



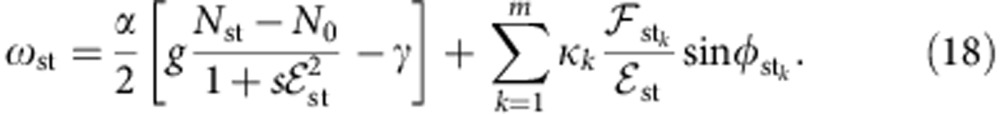


Substitution of equation (17) into equation (18) gives rise to


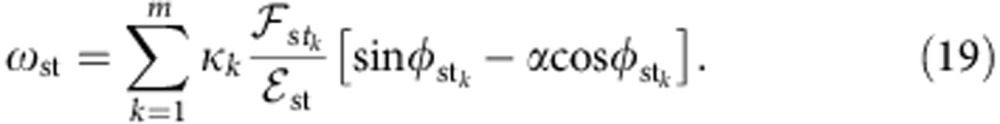


Writing the square braquet as


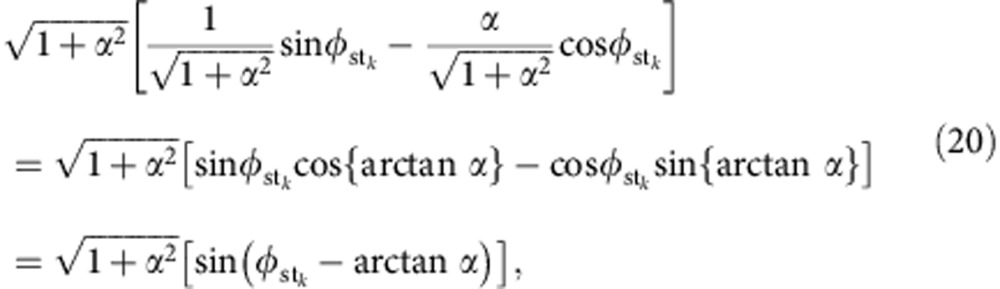


Equation (19) reads


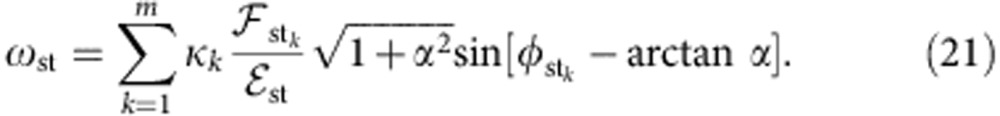


The 
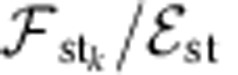
 dependence in equation (21) can be eliminated using equation (16),


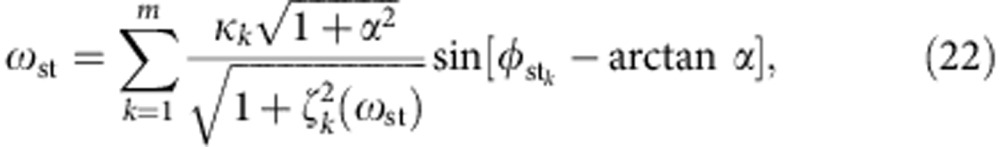


whereas inserting equation (15) into equation (22) we eliminate the *φ*_st_ dependence in equation (22) and get a transcendental equation for the frequency *ω*_st_:


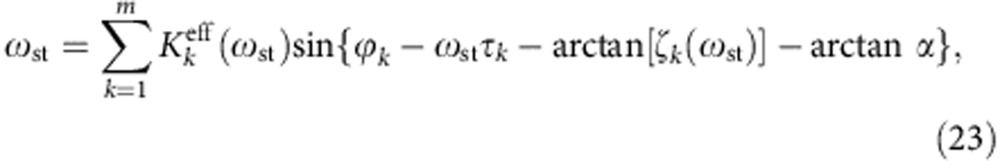


where,


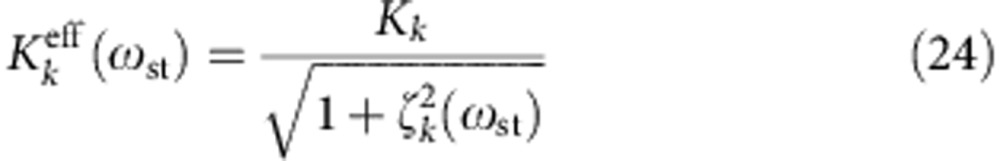


and 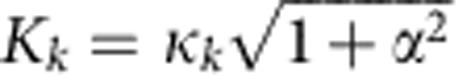 is the dressed feedback strength. In the case of a single filter (*k*=1) and 
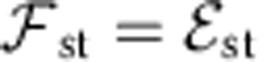
, which corresponds to conventional optical feedback (COF), this parameter is a measure of the number of relative equilibria of the system and it separates the region of monostable operation (*K*<1/*τ*) from the region of multistable operation (*K*>1/*τ*)^23^. Equation (23) can be solved numerically to find the frequencies of the relative equilibria. Each solution of equation (23) corresponds to the intersection of the straight line *ω*_st_ with the oscillating right-hand side of equation (23). It should be noticed that the argument of the sine is *ω*_st_ dependent itself, and it exhibits an additional *ω*_st_ dependence in the argument of the *arctan* function that is not present in COF. In the case of a single filter (*k*=1), the number of solutions of equation (23) increases with the effective feedback strength *K*^*eff*^(*ω*_st_), which is proportional to the feedback strength *κ*. As it can be deduced from equation (24), the number of relative equilibria with FFB is reduced as compared with the case of COF; nevertheless, for FFB with a single filter centred at *ω*_st_, that is, with Ω=*ω*_st_, equation (23) takes the same form that for COF. In general, for *s*≠0 the equations for carrier number *N*_st_ and the field amplitude 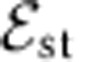 are coupled and one can not obtain a closed expression for them similar to Equation 22 for the frequency *ω*_st_. Nevertheless, taking *s*=0 one can obtain an approximation to the carrier number of the relative equilibrium with frequency *ω*_st_ from substitution of equations (11) and (15) into (17), 29,


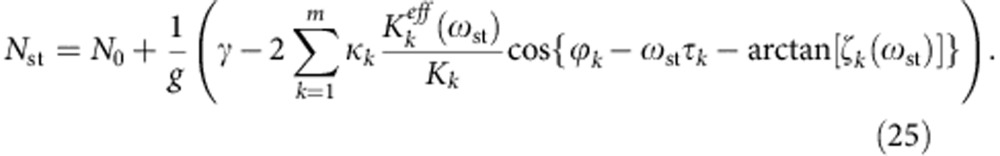


Taking *s*=0 will give rise to an approximative solution which is valid only for low laser intensities, as the case considered here. In this approximation, the field amplitude 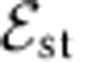 can be obtained from equation (9),


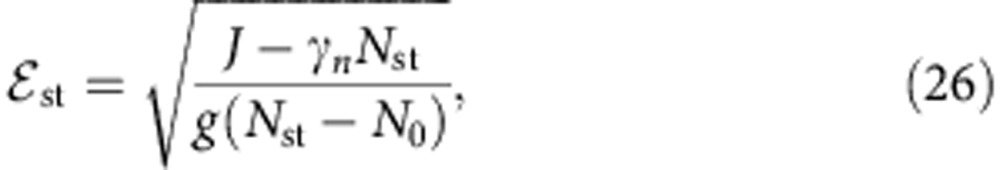


and 
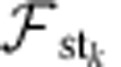
 can be derived from substitution of equation (15) into equation (11):


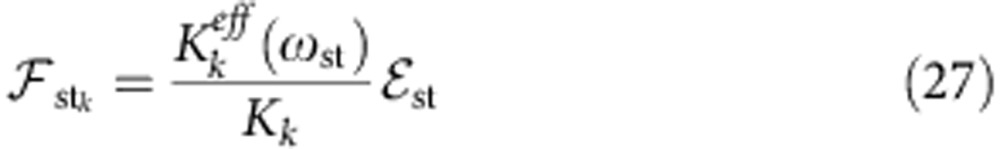


## Additional information

**How to cite this article**: Martnez-Llinàs, J. *et al.* Dynamical properties induced by state-dependent delays in photonic systems. *Nat. Commun.* 6:7425 doi: 10.1038/ncomms8425 (2015).
